# *N*‐Isopropylbenzylamine‐induced conditioned place preference, sensitization behaviour and self‐administration in rodents

**DOI:** 10.1111/adb.13370

**Published:** 2024-02-14

**Authors:** Miaojun Lai, Dan Fu, Xiangyu Li, Dingding Zhuang, Majie Wang, Zeming Xu, Huifen Liu, Haowei Shen, Peng Xu, Wenhua Zhou

**Affiliations:** ^1^ Department of Psychiatry Affiliated Kangning Hospital of Ningbo University Ningbo China; ^2^ Key Laboratory of Addiction Research of Zhejiang Province Ningbo China; ^3^ Office of China National Narcotics Control Commission China Pharmaceutical University Joint Laboratory on Key Technologies of Narcotics Control Beijing China; ^4^ Key Laboratory of Drug Monitoring and Control, Drug Intelligence and Forensic Center Ministry of Public Security Beijing China; ^5^ Faculty of Physiology & Pharmacology, School of Medicine Ningbo University Ningbo China

**Keywords:** abuse potential, drug use disorders, economic demand curve, *N*‐isopropylbenzylamine, psychomotor sensitization, psychostimulant

## Abstract

*N*‐Isopropylbenzylamine (*N*‐ipb), a chain isomer of methamphetamine (METH) with similar physical properties, has been used as a substitute for METH in seized drug samples. However, the abuse potential of *N*‐ipb remains unclear. Therefore, this study aimed to evaluate the abuse potential of *N*‐ipb in comparison to METH, by using conditioned place preference (CPP), locomotor sensitization and intravenous self‐administration tests. The results showed that *N*‐ipb at a dose of 3 mg·kg^−1^ significantly induced CPP in mice, which was comparable to the effect of METH at 1 mg·kg^−1^. Either acute or repeated *N*‐ipb injections (1 or 3 mg·kg^−1^) failed to raise the locomotor activity. However, acute treatment with 10 mg·kg^−1^ *N*‐ipb elevated the locomotor activity compared with saline, while chronic injection of 10 mg·kg^−1^ *N*‐ipb induced a delayed and attenuated sensitization compared with 1 mg·kg^−1^ METH. Rats could acquire *N*‐ipb self‐administration at a dose of 1 mg·kg^−1^·infusion^−1^, and a typical inverted U‐shaped dose–response curve was obtained for *N*‐ipb. The mean dose of *N*‐ipb that maintained the maximum response was greater than that of METH, indicating that *N*‐ipb is less potent for reinforcement than METH. In the economic behavioural analysis, comparison of essential values derived from the demand elasticity revealed that *N*‐ipb is less efficacy as a reinforcer than METH. The present data demonstrate that *N*‐ipb functions as a reinforcer and has a potential for abuse. However, the potency of psychomotor stimulation and the reinforcing effectiveness of *N*‐ipb are lower than those of METH.

## Graphical abstract

*N*‐Isopropylbenzylamine functions as a reinforcer and has the potential for abuse. The reinforcing potency and efficacy of *N*‐isopropylbenzylamine are both lower than those of methamphetamine.

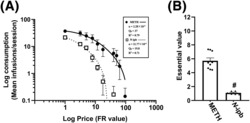

## 1 INTRODUCTION

Drug addiction is characterized by pathological neuroadaptation for the long term use of drug, accompanied with the emergence of aberrant behaviours, which can be assessed by using animal models such as drug place preference, behavioural sensitization and self‐administration. For example, repeated methamphetamine (METH) administration can induce behavioural sensitization,^1^, ^2^, ^3^ which has been suggested to underlie certain aspects of METH psychosis and schizophrenia.^4^ METH can elicit a positive reinforcement, which ultimately results into the abuse and addiction. The rodent conditioned place preference (CPP) model is one of the most popular drug reward paradigms and has been used widely to detect the rewarding effect of METH.^5^, ^6^ Moreover, the intravenous self‐administration model is considered the golden standard for evaluating the drug abuse potential, which has been used to assay not only the reinforcing effect of METH^7^ but also the potency^8^ and efficacy of METH reinforcement.^9^, ^10^, ^11^

**FIGURE 1:**
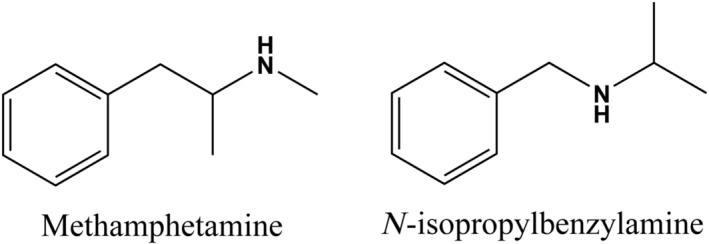
Chemical structures of methamphetamine (METH) and *N*‐isopropylbenzylamine (*N*‐ipb).

Since the early 2000s, club drugs (mostly new psychoactive substances) that can simulate the effects of illicit psychostimulants such as cocaine, methylenedioxymethamphetamine (MDMA) and METH have been widely used all over the world.^12^ In 2007–2008, Drug Enforcement Administration (DEA) reports have shown that a compound called *N*‐isopropylbenzylamine (*n*‐(1‐methylethyl)‐benzenemethanamin, *N*‐ipb, Figure 1) has been used first as an adulterant or substitute for METH in seized drug samples in the United States.^13^, ^14^, ^15^ *N*‐ipb is a chain isomer of METH (Figure 1), with the same chemical formula and molar mass. This similarity gives its hydrochloride salt a similar appearance and physical properties. *N*‐ipb has been used originally as an organic intermediate for pharmaceutical compounds and as a precursor for drug production.^16^ In New Zealand, many products of ‘pure METH’ sold in the street are adulterated with at least 50% of *N*‐ipb.^17^ Additionally, drug dealers in China have sold *N*‐ipb as fake ‘Ice’ METH in many recorded cases. Although *N*‐ipb is a border controlled substance in Australia, it is not currently known as a controlled substance in other countries.

For recreational purposes, the dosage of *N*‐ipb is approximately 25–100 mg per use dose, which is much higher than that of METH (5–30 mg per use dose), and users of *N*‐ipb have reported their experiencing brief stimulant effects, such as a rush or high, that are not typically associated with METH.^18^ These effects have been linked to side effects such as headaches and confusion that are not been commonly observed with METH use. In vitro experiments have also demonstrated that *N*‐ipb induces toxicity in neuron‐model cell lines.^19^ Despite a few clinical evidence and preclinical data, there is currently a lack of preclinical data characterizing the abuse potential of *N*‐ipb. To address this gap, we conducted this study by using rodent models such as locomotor sensitization, CPP and intravenous self‐administration to investigate the effects of *N*‐ipb on reward, psychomotor stimulation and reinforcement, respectively.

## 2 MATERIALS AND METHODS

### 2.1 Animals and drugs

Male SPF‐graded Sprague Dawley rats (weighing 290–320 g) were obtained from the Zhejiang Province Experimental Animal Center (Hangzhou, China), and male ICR mice (weighing 18–22 g) were purchased from SPF Biotechnology Co., Ltd. (Beijing, China). The rodents were individually housed in their home cages in a controlled room, with a reversed 12‐h light/dark cycle, a temperature set at 23 ± 2°C and a humidity level about 55 ± 10%. Food and water were provided without restriction in home cages. All experimental procedures were conducted in conformity to the Eighth Edition of the Guide for the Care and Use of Laboratory Animals.^20^ *N*‐ipb and METH were donated by the Drug Intelligence and Forensic Center, the Ministry of Public Security (Beijing, China), and each drug was dissolved in 0.9% sterile saline.

### 2.2 Conditioned place preference paradigm

The apparatus used in this study consisted of three Plexiglas chambers (40 × 15 × 36 cm^3^), a WAT‐902B HD camera and a computer. The two end chambers (17 × 15 × 36 cm^3^) were separated by a small central chamber (6 × 15 × 36 cm^3^) and were used for conditioning. The two larger chambers were visually and tactilely distinct: The right chamber consisted of 2 cm wide vertical black and white stripes on the walls with dot and stripe holes on the floor, while the left chamber had stripe‐free and round holes on the floor. The retention period in each chamber and the distance moved by the mice were recorded and calculated automatically by using ANY‐maze 6.0 (Stoelting, USA). The CPP paradigm was performed as described previously.^21^ The procedure comprised three phases: pre‐conditioning (day 1), drug conditioning (days 2–9) and post‐conditioning (day 10). On day 1, mice were habituated to the apparatus by being placed in the central compartment with the sliding doors opened, and the time spent in each chamber was recorded over 15 min. Mice with a residence time difference between the left and right chambers exceeding 120 s were excluded from further analysis. On days 2–9, eligible mice were divided randomly into five groups (*n* = 8–10): saline (saline, i.p.), METH (1 mg·kg^−1^, i.p.) and *N*‐ipb (0.3, 1, or 3 mg·kg^−1^, i.p.). At 9:00 AM on days 2, 4, 6 and 8, mice in each group receiving injections of METH or *N*‐ipb were immediately placed in the right chamber for a 45‐min conditioning session after each injection. At 9:00 AM on days 3, 5, 7 and 9, the same mice were injected with saline and then placed in the left chamber for another 45‐min conditioning session. On day 10, the mice were introduced to the central compartment without prior injection and given 15 min to explore the entire space freely. Their activity in each chamber was recorded via camera for 15 min in order to determine the CPP score for each group.

### 2.3 Locomotor sensitization

The locomotor sensitization test was conducted as previously described.^21^ All rats first adapted to the locomotor chambers with floors and walls made of clear acrylic and painted black (40 × 40 × 45 cm^3^) for 3 days (120 min/day). The rats were then divided randomly into five groups (*n* = 10): saline (saline, i.p.), METH (1 mg·kg^−1^, i.p.) and *N*‐ipb (1, 3, or 10 mg·kg^−1^, i.p.), and rats were tested for METH‐ or *N*‐ipb‐induced behavioural sensitization to locomotor activity for seven consecutive days (120 min/day). Two weeks of withdrawal after the last injection, following receiving saline or same drug, each rat was immediately placed in the locomotor chamber for 120 min. The locomotor activities of the rats were recorded and analysed using ANY‐maze 6.0.

### 2.4 Intravenous self‐administration experiments

The self‐administration training sessions were conducted using two standard nose‐poke operant conditioning cages (30 × 30 × 30 cm^3^) located within sound‐attenuated cubicles (Anilab SuperState, China). Two yellow lights inside nose pokes and a white house light on the ceiling of the chamber were all served as cue lights. A syringe pump, attached by means of a stainless‐steel swivel joint and connected to the rat, delivered drug infusions (0.047 mL·s^−1^) through Tygon tubing. The self‐administration training procedure was controlled by using the Anilab SuperState software (version 6.0; Ningbo, China).

#### 2.4.1 Surgery and catheterization

After acclimation for at least 7 days, rats were implanted with indwelling catheters in the right jugular vein under isoflurane anaesthesia as described previously.^22^ After surgery, the rats were allowed to recover for 7 days with daily injections of penicillin (60 000 units) and heparin (15 units) through catheters. Before and after the operant sessions, catheters were flushed daily with 0.3 mL saline and 0.3 mL heparinized saline (50 U·mL^−1^), respectively. From the second week, the catheter patency was further assessed by using 0.1 mL propofol solution (10 mg·mL^−1^) for sedation every three sessions.

#### 2.4.2 Dose–response determination

After recovery from surgery, all rats were randomized into three groups (*n* = 8). Rats of the METH and *N*‐ipb groups were initially self‐administered intravenously with METH under a fixed ratio 1 (FR1) reinforcement schedule, while rats of the saline group were subjected to self‐administration of saline. During the daily 4‐h self‐administration training session, illumination with a yellow light in the active nose poke hole served as a discriminative stimulus and signalled drug availability. Responses on the active hole resulted in an infusion of METH (0.05 mg·kg^−1^·infusion^−1^) or saline and initiated a 20‐s timeout during which the house light was illuminated for 5 s. Responding to the inactive hole or during a 20‐s timeout was recorded without other programmed consequences. METH self‐administration training was acquired and maintained for at least 10 days under these conditions, until the rats met acquisition criteria in the last three consecutive sessions (the range of variation in the mean number of infusions was within ±20%, more than 80% of total responses were on the active poke without upward or downward trend). Full dose–response curves were then generated for METH (0.0125–0.1 mg·kg^−1^·infusion^−1^), *N*‐ipb (0.25–0.2 mg·kg^−1^·infusion^−1^) and saline by dose substitution under the FR1 reinforcement schedule. The dosage ranges were selected based on the previous data of METH^10^ and *N*‐ipb.^19^ A counterbalanced order of doses was designed to obtain the complete dose–response functions, with at least one dose on the ascending limb, a peak dose and another dose on the descending limb. Each dose was evaluated for three consecutive sessions, then the original training dose was used for three sessions and the new dose was applied. All rats completed all parts of these trials.

#### 2.4.3 Behavioural demand curves

A behavioural economic demand procedure was used to evaluate the reinforcing effectiveness of *N*‐ipb and METH.^23^, ^24^ As described above, another batch of rats were prepared with intravenous catheters and trained to self‐administer METH (n = 8, 0.05 mg·kg^−1^·infusion^−1^), *N*‐ipb (n = 8, 1.0 mg·kg^−1^·infusion^−1^) or saline (n = 8) using an FR1 schedule. Consistent with the previous study, the training dose of METH or *N*‐ipb was the first dose on the descending limb of the above dose–response curves.^9^ Self‐administration training was maintained for at least 10 sessions when met the training stability standards for dose–response curve as described above. Once the training met the criteria, each FR value of the reinforcement schedule increased by 1.5 times that of the preceding response requirement. The following series of schedule values such as 3, 5, 8, 12, 18, 27, 41, 62, 93, 140, 210, 315 and so forth were used until no infusion occurred during a training session.

### 2.5 Data analysis

All data are presented as the mean ± standard error of the mean (SEM). The CPP scores were defined as the difference of time (s) spent in between the drug‐paired and the saline‐paired chambers. Locomotor sensitization was defined as a significant increase in locomotor activity after the third injection of drug compared with that after the first injection of drug.

Self‐administration acquisition data are presented as the number of active hole responses and the percentage of active hole responses over the total number of responses during the 10‐day sessions. For the METH self‐administration test, the difference in active responses per session for the two drug groups and the saline group was analysed by using paired *t*‐tests. The mean infusions for the last three sessions between the two drug groups were analysed by using paired *t*‐tests to examine the difference in total amounts in both groups during the training period. In the dose–response curve test, one‐way ANOVA was used to compare the difference of mean infusions earned for each dose of drugs across sessions with that in the saline group, followed by the Bonferroni *t*‐test for multiple comparisons. Reinforcement potency for each rat was measured by the unit dose that maintained the most responses on the dose–response curve and was indicated as the group mean ± 95% confidence interval as described before.^9^, ^10^

To analyse the reinforcing effectiveness of METH and *N*‐ipb, paired *t*‐tests were used to compare the mean infusions of either METH or *N*‐ipb with that of saline. According to the equation: log *Q* = log *Q*_0_ + *k (e* ^−α*Q*oC^ − 1),^25^ the log of the number of infusions was plotted as a function of log FR value, where *Q* is the mean number of infusions of each drug at FRx, *Q*_0_ is the mean infusions earned at FR1, C is the FR value and α, presented as a quantitative index of demand elasticity, is negatively correlated with reinforcing effectiveness and also could be transformed into an essential value (EV) according to the equation: EV = 1/(100αk^1.5^),^10^, ^26^ where *k* as a scaling constant was set to 4 in this study.^9^, ^10^ EV is directly correlated with the reinforcing effectiveness. *R* ^2^ as the correlation coefficient was calculated for each demand curve by using GraphPad Prism 8.0.1. *Q*_0_ was fixed as the mean infusions during the last three sessions earned under FR1. Differences in EV and *Q*_0_ for METH and *N*‐ipb were compared by using paired *t*‐tests.

## 3 RESULTS

### 3.1 *N*‐ipb induced CPP

**FIGURE 2:**
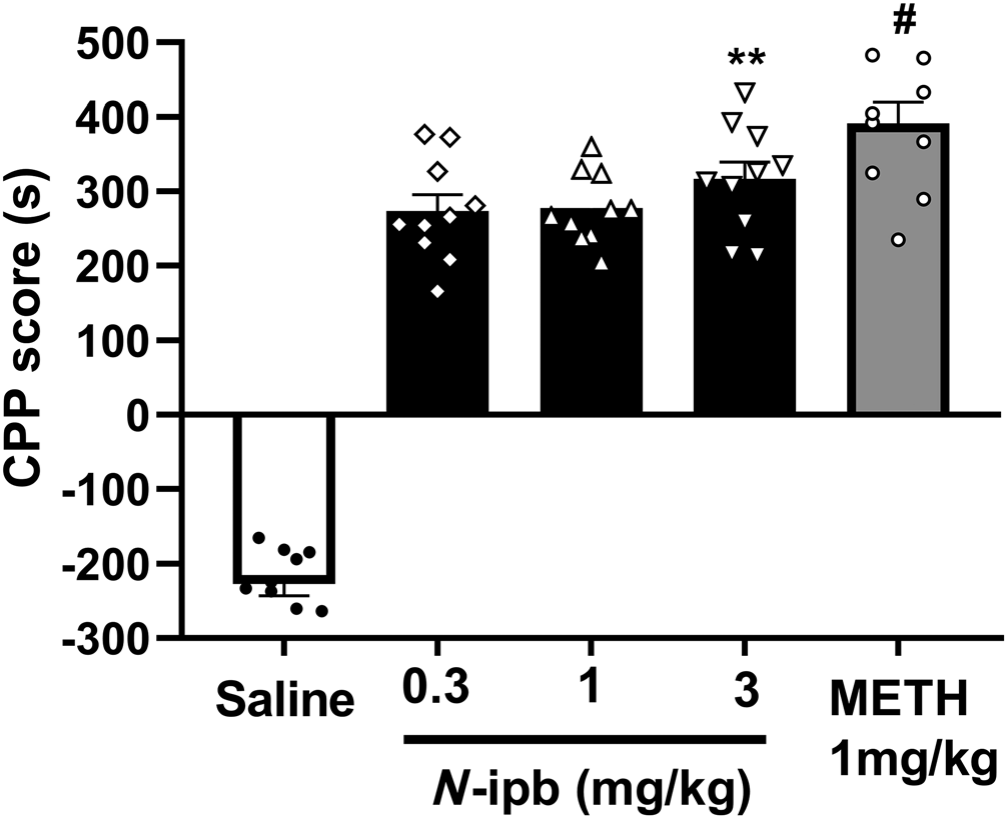
The comparison of methamphetamine (METH) and *N*‐isopropylbenzylamine (*N*‐ipb) conditioned place preference (CPP) in mice. CPP scores (the time spent in the drug‐paired compartment minus the time spent in the saline‐paired side) in mice on day 10 (post‐conditioning). **p < 0.01; ^#^ *p* < 0.001 compared with the saline group.

Mice were subjected to 5 days of CPP training during which they received either 1 mg·kg^−1^ of METH or 0.3, 1 or 3 mg·kg^−1^ of *N*‐ipb. On day 10, the CPP test was performed, and as illustrated in Figure 2, *N*‐ipb dose‐dependently induced CPP in the mice (one‐way ANOVA, *F*[3, 36] = 3.681, *p* = 0.021). The Bonferroni post hoc analysis revealed that the group of mice injected with 3 mg·kg^−1^ *N*‐ipb exhibited a significant preference for the drug‐associated chamber compared to the saline group (*p* < 0.01). Additionally, the *t*‐test showed that mice conditioned with 1 mg·kg^−1^ METH obtained significantly higher CPP scores than saline‐treated mice (*t* = 5.104, *p* < 0.001), but no significant difference was observed between the 1 mg·kg^−1^ METH‐treated group and 3 mg·kg^−1^ *N*‐ipb‐treated group (*t* = 2.058, *p* = 0.0543).

### 3.2 *N*‐ipb induced locomotor activities

**FIGURE 3:**
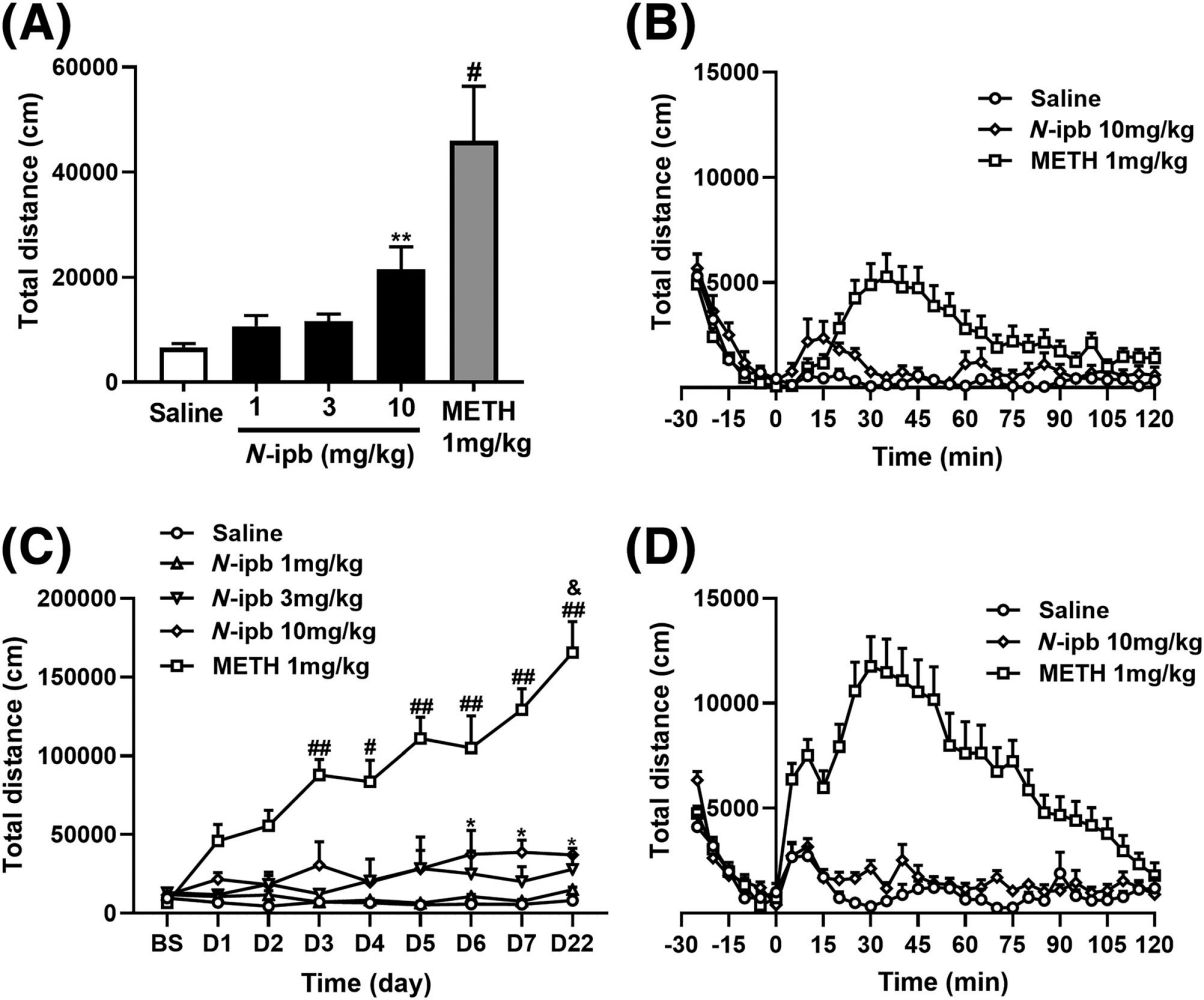
The effects of methamphetamine (METH) and *N*‐isopropylbenzylamine (*N*‐ipb) on the locomotor activity of rats. (A) Locomotor activity induced by acute injection of METH (1 mg·kg^−1^) or *N*‐ipb (1, 3 and 10 mg·kg^−1^) for 60 min on day 1, **p < 0.01, ^#^ *p* < 0.001 compared with the saline group. (B) The temporal pattern of locomotor induced by acute injection of METH (1 mg·kg^−1^) or *N*‐ipb (10 mg·kg^−1^) on day 1. Each point represents the average distance travelled in 5‐min bins. (C) Total distance during the 60‐min test on the baseline, each day of treatment and on the challenge day of METH, *N*‐ipb or saline, ^#^ *p* < 0.01, ^##^ *p* < 0.001 compared with day 1 (METH, 1 mg·kg^−1^); *p < 0.05 compared with day 1 (*N*‐ipb, 10 mg·kg^−1^); ^&^ *p* < 0.01 compared with day 7 (METH, 1 mg·kg^−1^). (D) The temporal pattern of locomotor response to acute METH (1 mg·kg^−1^) or *N*‐ipb (10 mg·kg^−1^) on the challenge day. Each point represents the average distance travelled in 5‐min bins.

Rats were injected chronically with METH (1 mg·kg^−1^) or *N*‐ipb (1, 3 or 10 mg·kg^−1^) once daily for seven consecutive days, followed by a 14‐day withdrawal period. On the first day of withdrawal, acute treatment with *N*‐ipb dose‐dependently produced an increase in locomotor activity (*F*[3, 44] = 6.66, *p* < 0.001). The Bonferroni post hoc analysis demonstrated that the rats injected with 10 mg·kg^−1^ *N*‐ipb exhibited a significant enhancement in locomotor activity relative to the saline group (*p* < 0.01, as shown in Figure 3A). The peak activity occurred at 10–20 min during the first 30 min after *N*‐ipb injection (Figure 3B). The *t*‐test showed that rats conditioned with 1 mg·kg^−1^ METH had significantly increased locomotion compared to saline‐treated rats (*t* = 3.757, *p* < 0.001, Figure 3A), with the peak activity at 25–45 min after injection (Figure 3B). Two‐way repeated‐measure ANOVA analysis of locomotor activities induced by chronic *N*‐ipb treatment indicated significant main effects of doses (*F*[3, 44] = 3.055, *p* = 0.0381) and days (*F*[8, 352] = 2.072, *p* = 0.0378), but not interactions between the doses and days (*F*[24, 352] = 1.311, *p* = 0.1516), and significant main effects of METH (*F*[1, 22] = 98.43, *p* < 0.0001), days (*F*[8, 176] = 16.79, *p* < 0.0001) and interactions between the factors (*F*[8, 176] = 17.35, *p* < 0.0001). Figure 3C showed that a statistically significant increase in locomotor activity on days 6, 7 and 22 (*p* < 0.05) was confirmed by using a Tukey's multiple comparison test for the group of rats administered 10 mg·kg^−1^ *N*‐ipb on day 1. In the group of rats administered 1 mg·kg^−1^ METH on day 1, a significant increase in locomotor activity was observed on days 3 and 5–22 (*p* < 0.001) as well as on day 4 (*p* < 0.01). Compared with day 7, 1 mg·kg^−1^ METH produced a significant increase in locomotor activity on day 22 (*p* < 0.01), whereas 10 mg·kg^−1^ *N*‐ipb did not. The within‐session locomotor activities induced by rechallenge with 10 mg·kg^−1^ *N*‐ipb and 1 mg·kg^−1^ METH on day 22 was shown in Figure 3D. The data indicated that a maximal dose of *N*‐ipb could enhance locomotor activity and induce delayed sensitization.

### 3.3 Dose–response curves and behavioural economics demand curves

**FIGURE 4:**
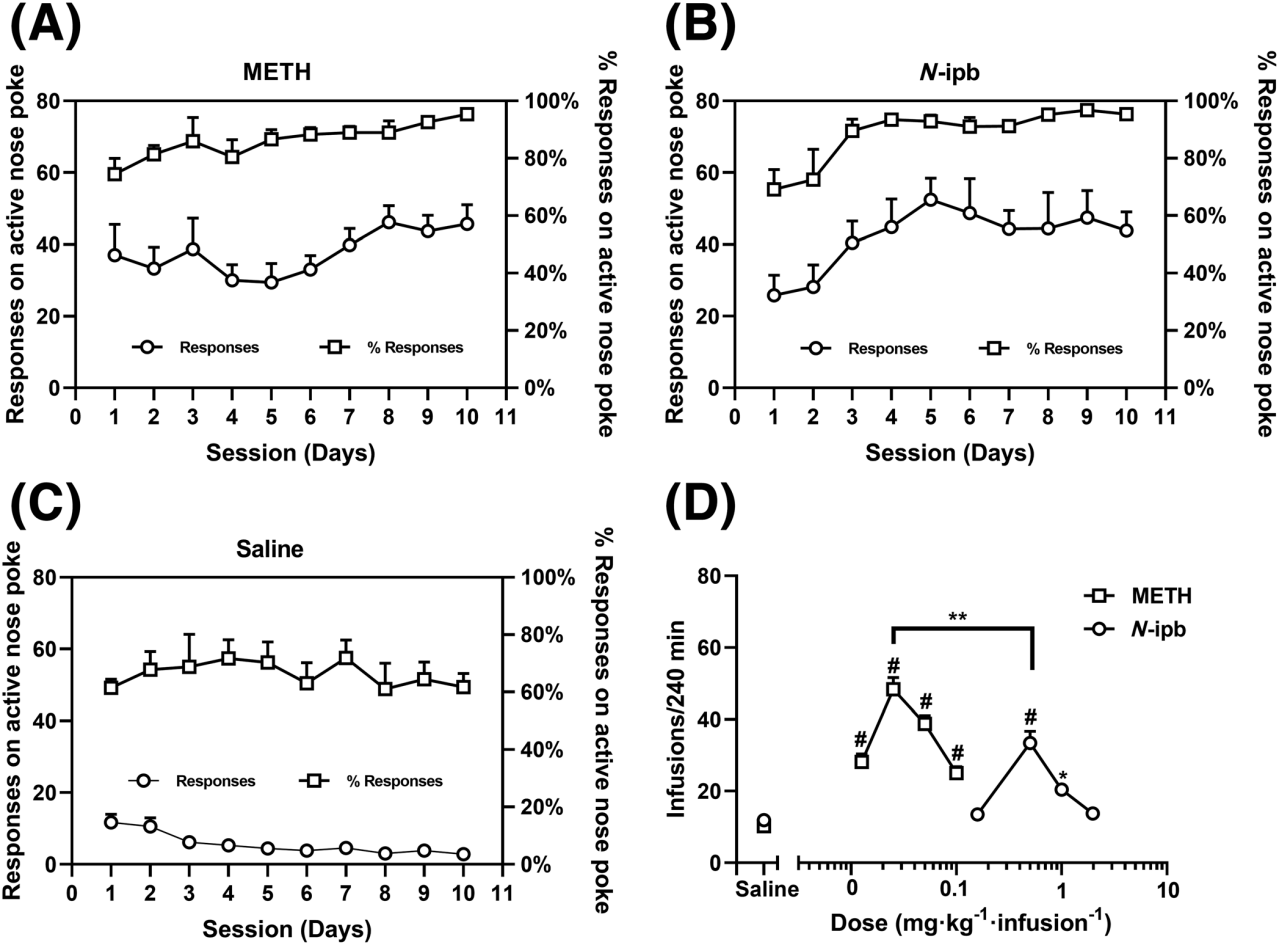
Data of self‐administration and dose–response curves for methamphetamine (METH) and *N*‐isopropylbenzylamine (*N*‐ipb). (A–C) Mean number of active responses and the percentage of active responses over total responses for the (A) METH and (B) *N*‐ipb groups of rats subjected to 0.05 mg·kg^−1^·infusion^−1^ METH self‐administration, compare with (C) those of saline groups (*n* = 8 per group). (D) Dose–response curves for the self‐administration of METH and *N*‐ipb under an FR1 schedule of reinforcement. *p < 0.05, ^#^ *p* < 0.01 compared with saline group.

As shown in Figure 4A–C, the levels of active nose‐poke responses to METH (0.05 mg·kg^−1^·infusion^−1^) were significantly higher than that to saline (METH group: *t* = 16.693, *p* < 0.001; *N*‐ipb: *t* = 15.366, *p* < 0.001), demonstrating that all rats in both drug groups readily acquired METH self‐administration under the FR1 schedule by the end of the 10‐day training session. No significant difference in the mean METH infusions during the last three sessions was observed between the METH and *N*‐ipb groups (*t* = 0.294, *p* = 0.771). The percentage of responses on active nose poke over the total number of responses on active and inactive pokes was greater than 80% for both drug groups, whereas the percentage of active responses in saline‐trained rats was less than 80%, as shown in Figure 4C.

Figure 4D depicted the dose–response curves for METH and *N*‐ipb. The curves displayed an inverted U‐shaped, indicating that both METH and *N*‐ipb produced bell‐shaped dose‐dependent effects. Notably, the dose–response curve for *N*‐ipb was shifted to the right compared to that for METH, suggesting that *N*‐ipb required a higher dose to produce a similar effect to METH. The mean number of infusions of METH or *N*‐ipb for each dose was significantly different from that of saline (METH: *F*[4, 100] = 42.561, *p* < 0.001; *N*‐ipb: *F*[4, 100] = 23.761, *p* < 0.001). The doses that maintained self‐administration significantly above the saline level for METH were 0.0125–0.1 mg·kg^−1^·infusion^−1^ (*p* < 0.001), while for *N*‐ipb, the doses were 0.5 (*p* < 0.001) and 1.0 mg·kg^−1^·infusion^−1^ (*p* < 0.05), respectively. METH and *N*‐ipb both maintained an average number of infusions/sessions above saline for at least one dose, indicating that both drugs acted as reinforcers in the rats. The mean dose (95% CI) that produced the maximum response (i.e., the peak dose) was 0.0316 (0.022, 0.041) mg·kg^−1^·infusion^−1^ for METH and 0.562 (0.470, 0.655) for *N*‐ipb, with rats earning on average 48.33 ± 3.33 and 33.43 ± 3.28 infusions, respectively, indicating that METH was a more potent reinforcer than *N*‐ipb in this procedure. A paired *t*‐test revealed that the mean number of infusions of METH at the peak dose was significantly greater than that of *N*‐ipb (*t* = 3.188, *p* < 0.01).

**FIGURE 5:**
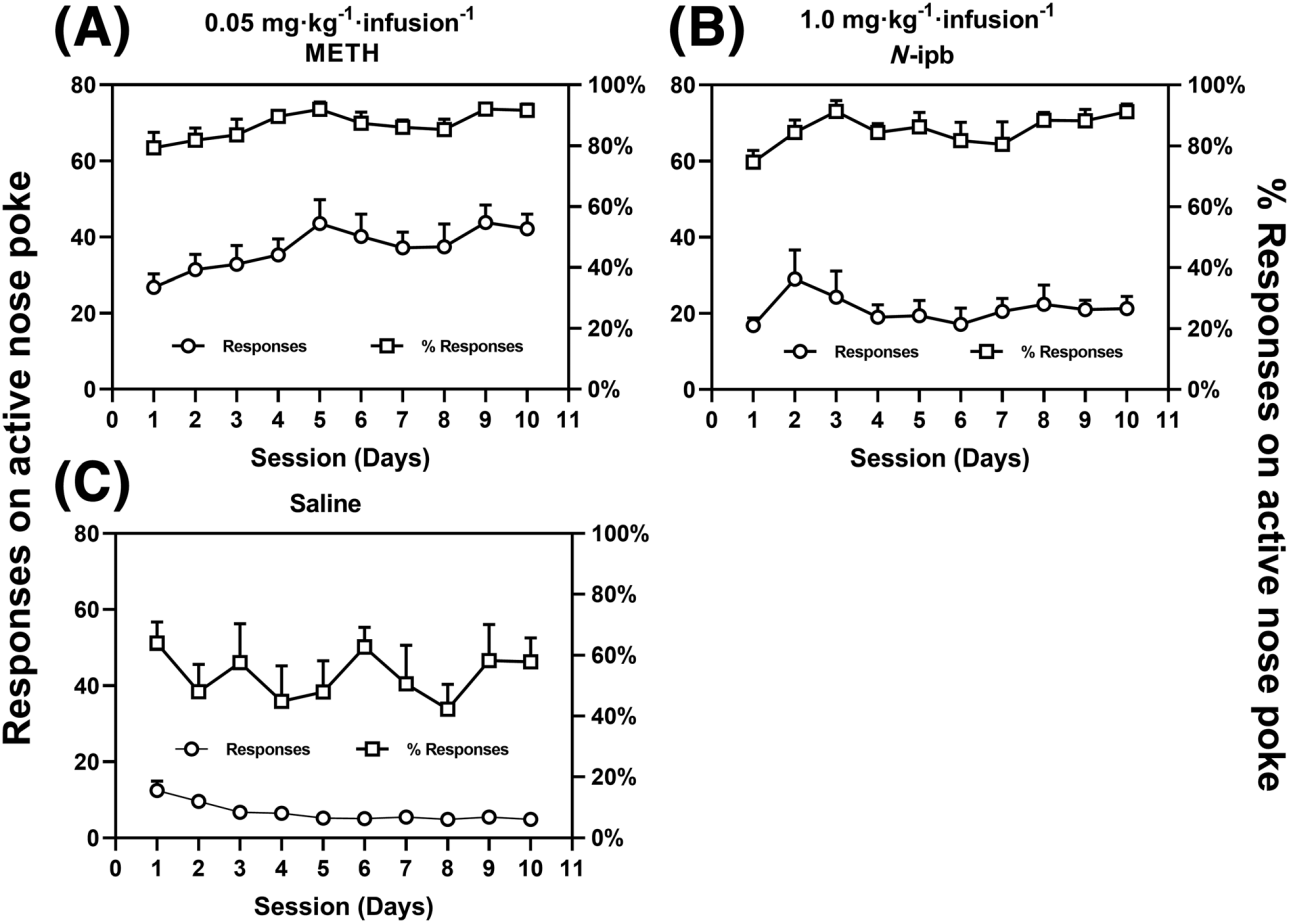
Mean number of active responses and the percentage of active responses over total responses for (A) 0.05 mg·kg^−1^·infusion^−1^ methamphetamine (METH), (B) 1.0 mg·kg^−1^·infusion^−1^ *N*‐isopropylbenzylamine (*N*‐ipb) and (C) saline during 10‐day training sessions of self‐administration in rats (*n* = 8 per group).

Only one rat was excluded from the behavioural economic determination due to its failure to respond during the *N*‐ipb training. All other rats in both drug groups readily acquired and maintained a response to the *N*‐ipb or METH for 10‐day training sessions. The number of active nose‐poke responses to either METH or *N*‐ipb across the sessions was significantly greater than that to saline (METH: *t* = 18.666, *p* < 0.001; *N*‐ipb: *t* = 9.762, *p* < 0.001; Figure 5). A two‐way repeated measures ANOVA revealed a significant main effect of the drugs (*F*[2, 18] = 55.53, *p* < 0.001), but not of the number of days (*F*[9, 162] = 0.5477, *p* = 0.8378), and a significant interaction between the factors (*F*[18, 162] = 1.769, *p* < 0.05) on active nose‐poke responses during the acquisition period. During the last three sessions, the percentage of responses to active nose‐pokes for METH or *N*‐ipb was consistently above 80%, while the percentage for saline was below 80%.

**FIGURE 6:**
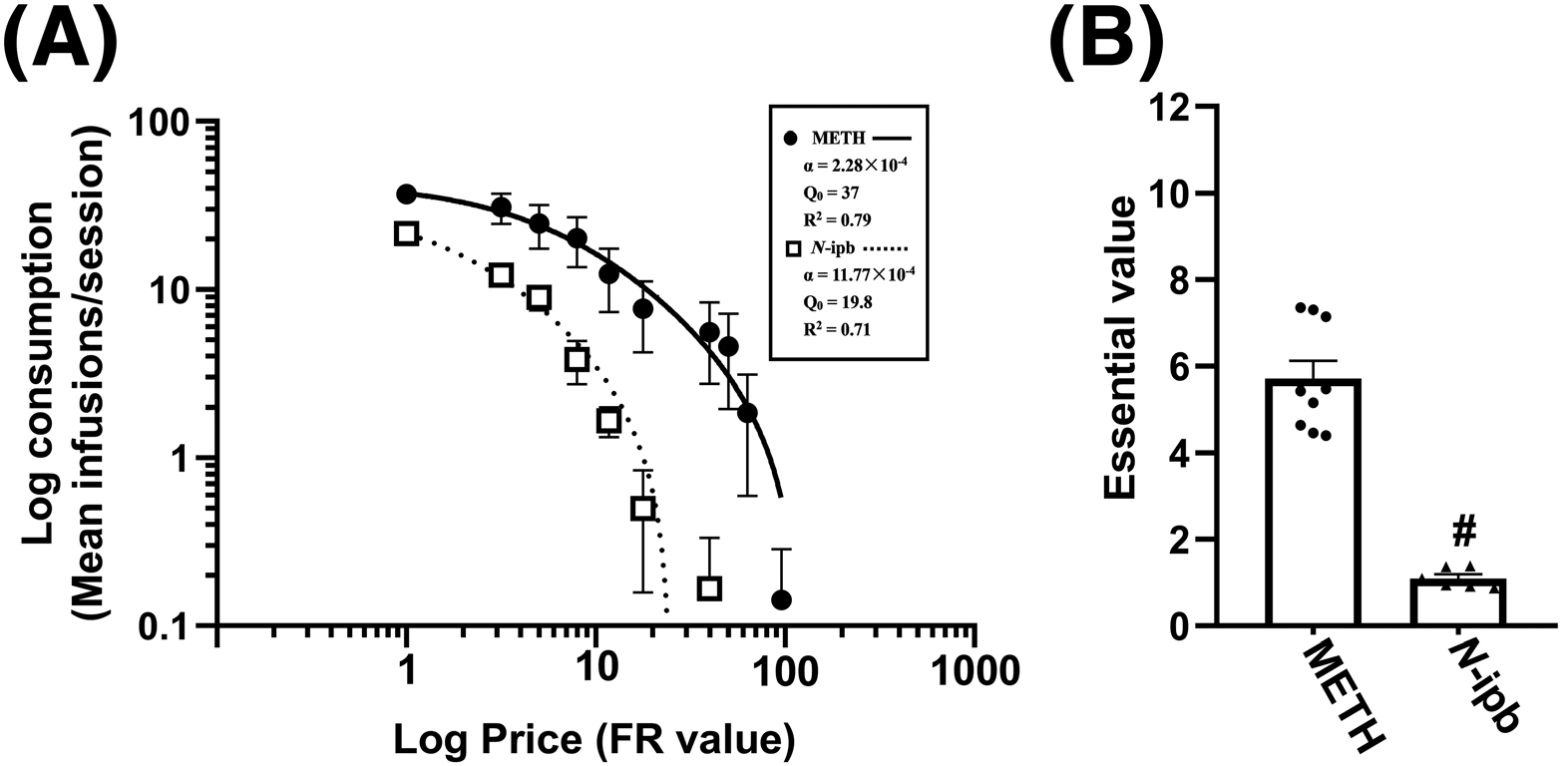
The demand curves and essential value of methamphetamine (METH) and *N*‐isopropylbenzylamine (*N*‐ipb). (A) Mean demand curves of self‐administration for METH (circles, *n* = 8) and *N*‐ipb (squares, *n* = 7). Abscissa: prices of METH and *N*‐ipb on a log scale. Ordinate: consumption expressed on a log scale. The α, Q_0_ and *R* ^2^ values for the two drugs are shown in the legend. Error bars represent the 95% confidence interval. (B) Mean essential values derived from the demand elasticity for each drug are plotted. Each data point indicates the mean essential value transformed from α for each FR of drugs. ^#^ *p* < 0.001 compared with METH.

In Figure 6A, the demand curves are presented as aggregated log consumption plotted against the log price for both METH and *N*‐ipb. The legends display the values of α, *Q*_0_, and *R* ^2^ for each drug. A paired *t* test revealed a significant difference between the METH and *N*‐ipb groups in *Q*_0_ (*t* = 4.873, *p* < 0.001). The demand elasticities (α) value of METH was equal to 0.000228 [0.000192–0.000263], while α value of *N*‐ipb was equal to 0.001177 [0.009368–0.001417]. Figure 6B showed the mean EV for METH and *N*‐ipb transformed from α, and the EV of METH (EV = 5.711 ± 0.411) was significantly higher than that of *N*‐ipb (EV = 1.099 ± 0.928; *t* = 10.961, *p* < 0.001). The results indicated that METH was more effective as a reinforcer than *N*‐ipb in this procedure.

## 4 DISCUSSION

The present research aimed to investigate the abuse potential of *N*‐ipb, which has a similar chemical structure and physical properties to those of METH. *N*‐ipb has been increasingly used as an adulterant or substitute for METH, raising concerns about its abuse potential. To assess this, we employed animal models of CPP, behavioural sensitization and drug self‐administration and compared the results with METH. This is the first study to evaluate the abuse potential of *N*‐ipb in comparison with METH, and our findings shed light on the potential risks associated with its use.

The CPP model is a well‐established paradigm for studying the rewarding effects of drugs and is commonly used to assess the abuse potential of new psychoactive substances in animals.^27^, ^28^, ^29^, ^30^ The present study found that *N*‐ipb dose‐dependently induced CPP, with a minimum effective dose of 3 mg·kg^−1^, whereas METH induced CPP in mice at a dose of 1 mg·kg^−1^ consistent with previous reports.^31^, ^32^ Behavioural sensitization has been defined as the aberrant progressive increase in locomotor activity when animals are exposed repeatedly to drug, implying a pathological form of neuroplasticity for drug addiction.^33^ Our results showed that *N*‐ipb exposure on the first day induced an increase in locomotor activity and led to the locomotor sensitization after 2 weeks of withdrawal at a dose of 10 mg·kg^−1^, whereas METH only at 1 mg·kg^−1^ could induce sensitization, consistent with a previous study.^34^ The peak activity induced by the acute injection of *N*‐ipb occurred earlier than that induced by METH, but the minimum effective dose of *N*‐ipb is higher than that of METH. Therefore, further studies should be combined with pharmacological experiments to determine whether *N*‐ipb has a more rapid onset of locomotor stimulation than METH. On the challenge day, compared with day 7, 1 mg·kg^−1^ METH induced a significant increase in locomotor activity, but 10 mg·kg^−1^ *N*‐ipb did not, indicating that METH is more potent than *N*‐ipb in psychomotor stimulation.

In the dose–response curve test, *N*‐ipb produced a typical inverted U‐shaped dose–response curve, and the mean dose of *N*‐ipb that maintained the maximum response was higher than that of METH, indicating that *N*‐ipb has a less potent reinforcing effect than METH. These findings are consistent with the results of the CPP test, where the minimum dose of *N*‐ipb required to induce CPP was about 3 mg·kg^−1^, which is also higher than the dose required for METH‐induced CPP. However, the tests of CPP, locomotor sensitization and the dose–response curve of self‐administration are not sufficient to predict the relative reinforcing efficacy of *N*‐ipb and METH. Here, we employed a behavioural economics procedure to assess the reinforcing efficacy of *N*‐ipb compared to that of METH. In this procedure, drugs with higher EVs are more likely to be abused than those with lower EVs, because EV is directly related to the efficacy of reinforcement.^25^ Our study revealed that the *N*‐ipb had a rightward shift of the curve and a decrease in EV, as indicated by the dose–response curve and the behavioural economics procedure respectively, suggesting that the abuse potential of *N*‐ipb is lower than that of METH. These findings may provide an explanation that *N*‐ipb is typically used at higher doses than METH for recreational purposes.^18^

METH is a widely abused psychostimulant that can cause persistent alterations in monoaminergic neuronal function, such as alterations in dopamine (DA) content, DA transporter (DAT) function and vesicular monoamine transporter‐2 (VMAT2) function/distribution.^35^, ^36^, ^37^, ^38^ In the self‐administration model, it has been reported that the reinforcing potencies of drugs acting on monoamine transporters are positively correlated with their affinity and potency at DAT,^39^, ^40^, ^41^, ^42^ whereas the measure of reinforcing effectiveness of drugs determined by the demand curve analyses is highly correlated with the DAT/serotonin transporter (SERT) uptake potency ratio.^9^, ^23^ The toxicity produced by *N*‐ipb in cell lines modelling neurons is related to the production of intracellular NO and the activation of nNOS.^19^ However, until now, the relative activities of *N*‐ipb at the different monoamine transporters are unknown, so further research is needed to confirm the affinity and potency of *N*‐ipb at monoamine transporters. It is also important to note that our study was conducted only in male mice or rats, and it is possible that the pharmacokinetics and pharmacodynamics of *N*‐ipb differ in females. Additionally, *N*‐ipb could substitute for the reinforcing effects of METH in the intravenous self‐administration but had a lower abuse potential than that of METH. It is reasonable to assume that *N*‐ipb may have a similar effect with METH when a mixture of *N*‐ipb and METH is used. As adulteration is common, the interactions or synergistic effects need to be further investigated when both substances are used together. We did not investigate whether *N*‐ipb fully substituted for the discriminative stimulus effect of METH in rats. Therefore, future experiments will include evaluations of the abuse potential of *N*‐ipb in female rodents and the subjective effects of *N*‐ipb or a mixture with METH.

In conclusion, our study provides the first evidence of the rewarding, psychomotor, and reinforcing effects of *N*‐ipb compared to METH in rodents, suggesting that *N*‐ipb has a potential for abuse. These findings highlight the importance of careful monitoring *N*‐ipb and timely regulation for control.

## AUTHOR CONTRIBUTIONS

Miaojun Lai, Peng Xu and Wenhua Zhou conceived the experiment. Miaojun Lai, Dan Fu and Xiangyu Li conducted all the experiments. Miaojun Lai, Peng Xu, Majie Wang and Dingding Zhuang analysed the results. Miaojun Lai, Huifen Liu, Zeming Xu, Haowei Shen and Wenhua Zhou wrote the manuscript, and all authors reviewed the manuscript.

## CONFLICT OF INTEREST STATEMENT

The authors declare no conflict of interest related to this work. Each author has indicated that he/she has met the journal's requirements for authorship.

## Data Availability

The data used to support the findings of this study are available from the first author and corresponding authors upon request.
